# GPU-accelerated and pipelined methylation calling

**DOI:** 10.1093/bioadv/vbac088

**Published:** 2022-11-30

**Authors:** Yilin Feng, Gulsum Gudukbay Akbulut, Xulong Tang, Jashwant Raj Gunasekaran, Amatur Rahman, Paul Medvedev, Mahmut Kandemir

**Affiliations:** Department of Computer Science and Engineering, The Pennsylvania State University, University Park, PA 16802, USA; Department of Computer Science and Engineering, The Pennsylvania State University, University Park, PA 16802, USA; Department of Computer Science, University of Pittsburgh, Pittsburgh, PA 15260, USA; Adobe Research, Adobe, San Jose, CA 95110, USA; Department of Computer Science and Engineering, The Pennsylvania State University, University Park, PA 16802, USA; Department of Computer Science and Engineering, The Pennsylvania State University, University Park, PA 16802, USA; Department of Biochemistry and Molecular Biology, The Pennsylvania State University, University Park, PA 16802, USA; Huck Institutes of the Life Sciences, The Pennsylvania State University, University Park, PA 16802, USA; Department of Computer Science and Engineering, The Pennsylvania State University, University Park, PA 16802, USA

## Abstract

**Motivation:**

The third-generation DNA sequencing technologies, such as Nanopore Sequencing, can operate at very high speeds and produce longer reads, which in turn results in a challenge for the computational analysis of such massive data. *Nanopolish* is a software package for signal-level analysis of Oxford Nanopore sequencing data. Call-methylation module of *Nanopolish* can detect methylation based on Hidden Markov Model (HMM). However, *Nanopolish* is limited by the long running time of some serial and computationally expensive processes. Among these, Adaptive Banded Event Alignment (ABEA) is the most time-consuming step, and the prior work, *f5c*, has already parallelized and optimized ABEA on GPU. As a result, the remaining methylation score calculation part, which uses HMM to identify if a given base is methylated or not, has become the new performance bottleneck.

**Results:**

This article focuses on the call-methylation module that resides in the *Nanopolish* package. We propose *Galaxy-methyl*, which parallelizes and optimizes the methylation score calculation step on GPU and then pipelines the four steps of the call-methylation module. *Galaxy-methyl* increases the execution concurrency across CPUs and GPUs as well as hardware resource utilization for both. The experimental results collected indicate that *Galaxy-methyl* can achieve 3×–5× speedup compared with *Nanopolish*, and reduce the total execution time by 35% compared with *f5c*, on average.

**Availability and implementation:**

The source code of *Galaxy-methyl* is available at https://github.com/fengyilin118/.

## 1 Introduction

To decrease the cost of genome sequencing to promote genomics research, several third-generation sequencing technologies such as Nanopore Sequencing (Simpson *et al.*, 2017) have recently been developed. To speed up these technologies, several portable sequencing devices that allow the detection of epigenetic states of cells have been created. Analyzing these epigenetic changes across many genes in a cell or an organism is known as *epigenomics* (National Cancer Institute, 2022).

DNA methylation is an important aspect of epigenome, and therefore, detecting methylation is a critical operation for the regulation and maintenance of important cellular activities. Most importantly, methylation detection plays an important role in human disease diagnosis such as cancer (Bichindaritz *et al.*, 2021), diabetes (Martorell-Marugán *et al.*, 2019) and immune system-related disorders (Calle-Fabregat *et al.*, 2020). DNA methylation is an occurrence of methyl groups in a DNA molecule.

Methylation detection (methylation calling) is a compute- and storage-intensive process. It consists of four steps: *event detection*, *alignment*, *final calibration* and *methylation score calculation*. Each of these steps is associated with different degrees of parallelism. Several prior works such as *Nanopolish call-methylation* (Simpson *et al.*, 2017) and *f5c* (Gamaarachchi *et al.*, 2020) have parallelized the different steps of the methylation calling. *MultiNanopolish* (Hu *et al.*, 2021) uses multi-threading to decompose the iterative calculation in *Nanopolish* into small independent calculation tasks to run in parallel mode. However, *Nanopolish* and *MultiNanopolish* primarily employ CPU-based parallelization and *f5c* only uses GPU to parallelize a certain task within methylation calling. Among the different implementations of methylation calling (Gamaarachchi *et al.*, 2020; Hu *et al.*, 2021; Simpson *et al.*, 2017), *Nanopolish* is a widely used software tool for genomic sequencing. *f5c* is the modified version of the popular methylation detection tool *Nanopolish*. *f5c* (Gamaarachchi *et al.*, 2020) parallelizes the Adaptive Banded Event Alignment (ABEA) process on GPUs. However, little attention has been given to the methylation score calculation process, which occupies a large fraction of the execution time in methylation detection. We observe that the methylation score calculation can be *parallelized* and compute-accelerators such as GPUs can be leveraged to achieve significant speedups. Motivated by this, we modify *f5c* with the goal of achieving a more efficient use of the CPU and GPU resources through code parallelization.

Oxford Nanopore Technologies also released Remora (Nanopore, 2022), which is a lightweight methylation calling model and is recommended to run on CPU resources. Remora separates methylation base calling from base calling neural network, achieving higher quality results from simpler training samples. Remora employs a machine learning (ML)-based method to perform binary or categorical prediction for modified base content of nanopore reads, while our work utilizes GPU to accelerate HMM to distinguish 5-methylcytosine from unmethylated cytosine. Furthermore, data preparation is needed to produce chunks in a Remora-friendly format for training and Remora then produces a final model in ONNX format. Finally, the inference module within Remora uses the model for testing the input dataset, which is the Taiyaki-mapped signal file. It is to be noted that the input and output data format is different from that of the Nanopolish Call-methylation module.

GPUs have been dominating the hardware-accelerator landscape due to their high potential of supporting modern-day applications (Gudukbay *et al.*, 2021; Jiang *et al.*, 2020; Narayanan *et al.*, 2021). As the programmability of GPUs has rapidly increased over years, the compute and storage capabilities of GPUs have increased significantly, allowing them to meet the challenging parallelization requirements imposed by many different domains including bioinformatics. Thus, several bioinformatics applications that are critical for human lives have been successfully accelerated (Baek *et al.*, 2021; Dee, 2022; MacCarthy *et al.*, 2022) using GPUs. Our work is motivated by the high degrees of intrinsic parallelism exhibited by methylation calling that can potentially be harnessed by GPUs.

By utilizing the capabilities of GPUs to accelerate methylation calling, in this article, we make the following key *contributions*:

- We identify substantial parallelism opportunities in the methylation score calculation step of methylation calling, which was not addressed by prior works (Gamaarachchi *et al.*, 2020; Hu *et al.*, 2021; Simpson *et al.*, 2017). Specifically, this step employs a Hidden Markov Model (HMM) algorithm to calculate methylation scores for a matrix, thereby presenting significant parallelization opportunities thanks to the lack of data dependencies.
- We implement a GPU-parallelized version of the methylation scoring task of methylation calling, which we refer to as *Galaxy-methyl*. Our extensive experimental analysis reveals that this implementation leads to both CPU and GPU underutilization.
- To solve this resource underutilization problem, we propose and implement a *pipelined execution* for *Galaxy-methyl* that increases concurrency, thus improving both the GPU and CPU hardware resource utilization.
- We collect experimental data to demonstrate the effectiveness of our approach. The experimental results indicate that the proposed GPU implementation outperforms the CPU-based implementation of methylation score calculation by ∼2×. Furthermore, by combining the parallel ABEA execution proposed by *f5c* (Gamaarachchi *et al.*, 2020) with our GPU methylation score calculation, *Galaxy-methyl* can achieve 35% speedup in total execution time compared with running only *f5c*, and 3×–5× speedup compared with *Nanopolish*.
- We add *Galaxy-methyl*, as a new tool, into the Galaxy framework (Community, 2022), to facilitate researchers to detect DNA methylation with high performance.

## 2 Background

In this section, we explain (i) the details of Nanopolish software package and (ii) the HMM algorithm which is a key component of methylation calling.

### 2.1 Nanopolish call-methylation module

**Fig. 1.**
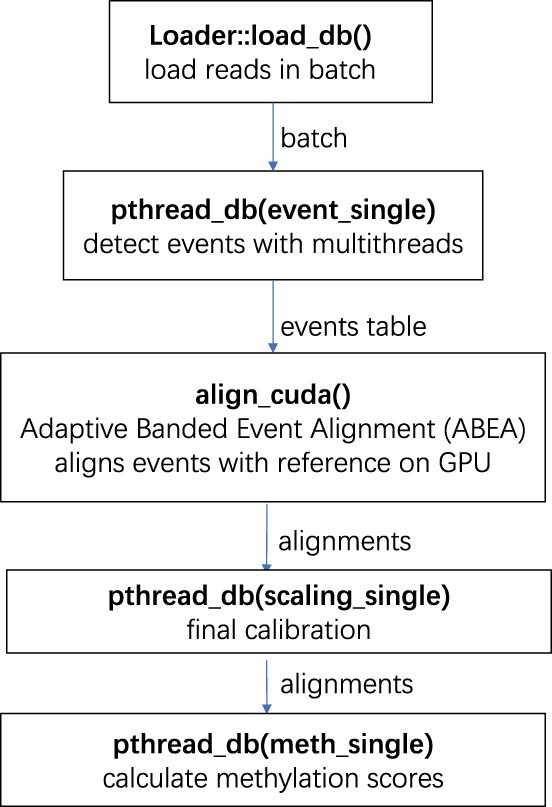
Call-methylation flow chart

*Nanopolish* is a software package for signal-level analysis of Oxford Nanopore Sequencing data. It utilizes the raw signals to improve the accuracy of error correction in the draft assembly. It has four modules: *call-methylation*, *variants*, *consensus* and *eventalign*. We use the call-methylation module of the *Nanopolish* package as a *baseline* for our proposed version of methylation calling. As depicted in Figure 1, for a given read, the call-methylation module works in four steps: (1) event detection; (2) alignment; (3) final calibration and (4) methylation score calculation. The first step is to detect events from raw signals using time-series segmentation based on sudden signal level changes. Segments, which are the output of this segmentation process, are called *events*. The detected events are aligned to a generic k-mer model signal to obtain the true annotation using the *ABEA* algorithm in the second step. The third step is called *final calibration* and performs recalibration to get the best set of scaling parameters for the alignments. The last step, which is the *calculation of methylation scores*, uses HMM (It is a statistical Markov model where the system being modeled is assumed to be a Markov process with ‘unobservable’ states.) to calculate the likelihood of the unmethylated sequence and methylated sequence to detect methylation (Simpson *et al.*, 2017).

The *Nanopolish* call-methylation module uses a modified Viterbi algorithm for HMM to calculate the likelihood of derived haplotype sequences. There exist prior works that implement the Viterbi algorithm for HMM on GPUs. For example, Horn *et al.* (2005) present an HMM-based sequence alignment on GPUs that uses the streaming Viterbi algorithm. On the other hand, a tile-based parallel Viterbi algorithm (Du *et al.*, 2010) fills a matrix with wave-front pattern to take advantage of the computing power of the GPU. In comparison, CUDAMPF (CUDA Multi-Tiered Parallel Framework) (Jiang and Ganesan, 2016) is a multiple-tiered parallel framework for finer-grained parallel Viterbi algorithms, comprising of Single Instruction, Multiple Thread (SIMT)/Single Instruction, Multiple Data (SIMD) execution. Cheng and Butler (2015) optimize Multiple Segment Viterbi in HMMER3 (Finn *et al.*, 2011) on GPUs, using SIMD video instructions, minimizing and coalescing global memory access, using asynchronous memory copy and streams. Since the *Nanopolish* module uses a modified version of Viterbi algorithm for HMM, these previous GPU implementations are unfortunately not directly applicable. In particular, the wave-front pattern parallelization in Du *et al.* (2010) cannot solve the data-dependence on the upper-right cell in the probability matrix in *Nanopolish*. Also, in Cheng and Butler (2015), each thread processes a different neighboring sequence. This approach cannot be directly used in *Nanopolish* since it may lead to overloaded threads and thread load imbalance. Jiang and Ganesan (2016) calculate one scoring matrix for each sequence whereas *Nanopolish* calculates one scoring matrix for each CpG groups.

### 2.2 HMM algorithm

**Fig. 2.**
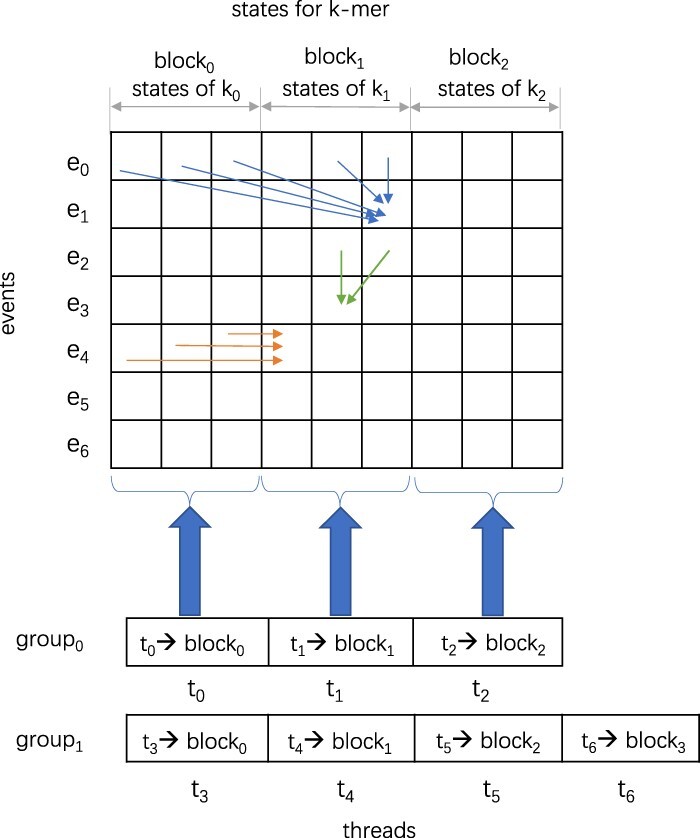
Thread assignment of HMM

In *Nanopolish*, HMM calculates the probability of the alignment by filling a probability matrix. Figure 2 illustrates the filling process and Algorithm 1 gives the corresponding algorithm.

Algorithm 1: HMM.

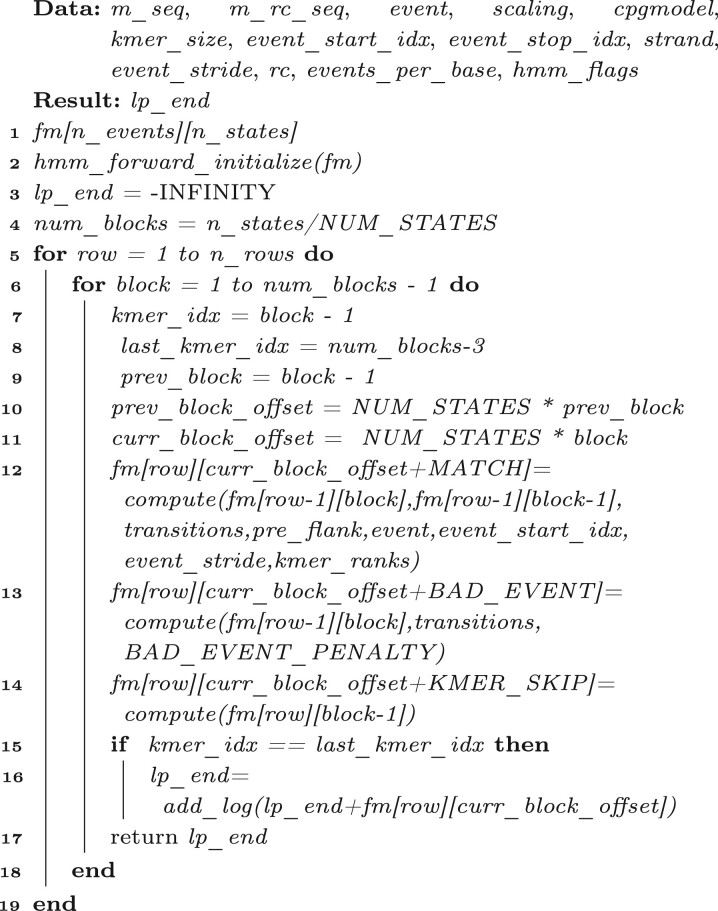

Figure 2 shows each cell in the matrix storing the probability of the alignment ending at that specific event (row) and state (column). Each k-mer has three states (*KMER_SKIP*, *BAD_EVENT* and *MATCH* in order), and they collectively form one block. The matrix is updated from the first row to the last row and from the first block to the last block (lines 5–19 in Algorithm 1). The blue arrow in Figure 2 shows that the probability at (*row-1*, *block-1*) and (*row-1*, *block*) is used to compute the probability at *fm[row][curr_block_offset + MATCH]* (line 12 in Algorithm 1). The green arrow indicates that *fm[row][curr_block_offset + BAD_EVENT_PENALTY]* is updated using the probability at (*row-1*, *block*) (line 13 in Algorithm 1). The orange arrow shows that the probability at (*row*, *block-1*) is used to update *fm[row][curr_block_offset + KMER_SKIP]* (line 14 in Algorithm 1). When the last block is updated, the *add_log* function is used to add the probabilities of the alignment ending at current row and block (lines 15 and 16 in Algorithm 1). The log probability for the final state is returned as the output.

## 3 Methods

In this section, we explain the practical need for accelerating methylation calling and discuss the details of *Galaxy-methyl*.

### 3.1 Opportunities for improvement and challenges

This work focuses on accelerating the *Nanopolish* call-methylation module (Simpson *et al.*, 2017). The methylation score calculation task of the methylation calling module is a significant, time-consuming part of the whole application, and its crucial algorithmic component is a modified version of HMM. HMM is used to calculate the probability of observing a sequence of signal-level events given a specific nucleotide sequence to detect methylation. The probability calculation is implemented by filling probability matrices, whose cells are probabilities of ending the alignment at that specific event (row) and state (column) of k-mers of the reference genome. HMM is a compute-intensive process that can significantly benefit from GPU acceleration for reducing the execution time of the call-methylation module, considering the increasing amount of data generated by the third-generation sequencing technology. Due to the data dependencies and underutilization challenges that come from the HMM algorithm, the methylation score calculation task has not been parallelized in the previous work. For example, *f5c*, a recent GPU-implementation based on *Nanopolish*, does not focus on this part, and only parallelizes and optimizes the ABEA algorithm on GPU. Table 1 shows *f5c*’s processing time of each step on a sample dataset. It can be seen that the methylation score calculation step (HMM) takes about 25% of the total execution time when ABEA is implemented on GPU. To our knowledge, this step has not been parallelized in any of the previous works.

**Table 1.** f5c processing time

Total time (s)	Events detection (s)	ABEA (s)	Calibration (s)	HMM (s)
694	178	320	21	175

Thus, there are big opportunities to optimize the methylation score calculation step to reduce the total execution time of the call-methylation module. The crucial component of this step is the HMM implementation. Due to the massively parallel probability calculation involved in it, GPU seems to be a very promising compute-platform to accelerate the HMM. Furthermore, as will be explained later, implementing the methylation score calculation step on GPU can further facilitate the ‘pipelined execution’ of the whole call-methylation module, thereby increasing hardware resource utilization and ultimately improving the overall application performance.

GPU implementation of methylation score calculation is not straightforward due to several challenges. The first of these challenges is the extensive use of complex data structures such as vectors or strings in the original *Nanopolish* call-methylation module and the unavailability of those data structures in GPU programming paradigms/frameworks. More specifically, in the CUDA framework (which is used extensively for GPU programming), complex data structures are not directly supported.

The second challenge is that the probability calculation in HMM has some ‘data dependencies’; consequently, it is not a straightforward task to parallelize HMM on GPUs. HMM calculates the probability values by filling the probability matrices whose cells are the probabilities of the alignment ending at that specific event (row) and state (column) of k-mers of the reference genome. As a result, each cell of the probability matrix in the HMM algorithm is *dependent* on the previously seen probabilities in the same matrix. Figure 2 shows the data dependencies of probability computation in the probability matrices. Each k-mer has three states, namely, *PSR9_MATCH*, *PSR9_BAD_EVEN* and *PSR9_KMER_SKIP*. The blue arrow indicates that the probability calculation of the alignment ending at *event*_1_ and *PSR9_MATCH* of *k-mer*_1_ depends on the probability of the alignment at previous event *event*_0_ and states of *k-mer*_0_, *k-mer*_1_. The green arrow illustrates that the probability of the alignment ending at *event*_3_ and *PSR9_BAD_EVEN* of *k-mer*_1_ depends on that at previous event *event*_2_ and states of *k-mer*_1_. Similarly, the orange arrow shows that the probability of the alignment ending at *event*_4_ and *PSR9_KMER_SKIP* of *k-mer*_1_ depends on that at current event *event*_4_ and states of the previous *k-mer*.

Another challenge is that, in the *Nanopolish* call-methylation module, one batch of the input dataset is processed at a time and each task of each batch is processed serially (one task after another). Consequently, since both the alignment step and the methylation score calculation step are processed only on the GPU, the CPU remains idle while GPU performs calculations. Similarly, when event detection and dataset loading are performed on the CPU, the GPU remains idle.

To address these challenges, we propose the following approaches. For solving the challenge related to the lack of dynamically allocated complex data structures in GPU programming, we allocate a fixed-sized memory by approximately estimating its size a priori and use auxiliary arrays to save the starting index or used size for each read. If some unsupported data structures are used, we transfer them to arrays that are supported by the CUDA framework. For example, *string* data structures are transferred to *char* arrays. Then, we construct three states of one k-mer into one block and we divide the matrix-filling step of HMM into two parts. The first part is block-independent; so, blocks can be processed in parallel. The other part is block-dependent; so, we serially process blocks, which means only one thread is selected to process all blocks for one read. This solves the challenge related to data dependencies. Finally, we *pipeline* four steps of call-methylation module to utilize both CPU and GPU efficiently, and this solves the challenge due to resource idleness/underutilization.

### 3.2 Acceleration of call methylation

This section describes our GPU-based optimization method—*Galaxy-methyl*. Since HMM is a crucial component of the methylation score calculation step as explained earlier in Section 2.2, we profiled the call-methylation module using NVProf (https://docs.nvidia.com/cuda/profiler-users-guide/index.html) to (i) understand how much of the total execution time the HMM task consumes and (ii) identify the architectural bottlenecks. Our profiling shows that HMM occupies nearly 70% of the methylation score calculation execution time; hence, it is the primary bottleneck in the methylation score calculation step. Next, we present our GPU implementation for optimizing and parallelizing HMM along with the proposed pipeline optimization for the whole call-methylation model.

### 3.3 GPU implementation

The HMM algorithm is applied to all CpGs groups in all reads; as a result, it is embarrassingly parallel. Since GPUs use the SIMD paradigm (SIMD denotes an organization of parallel execution where multiple compute-units operate, under the supervision of a common control unit, on different data.), they are suitable to implement and execute the HMM algorithm. However, the GPU implementation for the HMM is non-trivial, due to the dependencies among cells in the probability matrix, as explained before in Section 2.2.

In our implementation, the probability matrix is divided into multiple blocks; each block includes the probabilities of the alignment ending at events and three states of one k-mer. Figure 2 shows the thread assignment for HMM on GPU. Each block is assigned to one thread. For example, three threads are assigned to three blocks of the first group and four threads are assigned to four blocks of the second group. Note that the threads assigned to one group should be in the same thread block for block synchronization in CUDA. Therefore, updating the probabilities of the alignment at states *PSR9_MATCH*, *PSR9_BAD_EVEN* can be done for each block in parallel since that is block-independent, as blue and green arrows show. However, the probability of alignment at state *PSR9_KMER_SKIP* is dependent on the previous block. As a result, just the first thread in each group is assigned to update this probability serially, for example, threads *t*_0_ and *t*_3_ in Figure 2. Algorithm 2 gives the kernel function which is assigned to each thread. First, it obtains the *thread_id*, *block_id*, number of blocks *n_block* and number of rows *n_row* from line 1 to line 4. After that, it iterates from the first row to the last one to fill the probability of the alignment at the current row and states of the current block. Like Algorithm 1, it fills *fm[row][curr_block_offset + MATCH]* at line 12 and *fm[row][curr_block_offset + BAD_EVENT]* at line 13 which are the probability of the alignment at current row and the state *PSR9_MATCH* and *PSR9_BAD_EVENT*, respectively. The filling for the two is dependent on *fm[row-1]*, which is the probability at the previous row and has already been calculated. Hence, the two probability calculations are block-independent and can be done in parallel. Following that, it synchronizes threads in one thread block. Next, it fills the probability of the alignment at the current row and the state *PSR9_KMER_SKIP* from lines 15 to 18. It is to be noted that, since the computation of *fm[row][curr_block_offset + KMER_SKP]* depends on the probability of the previous block, it is filled sequentially. HMM is applied to all CpGs groups in all reads. Therefore, only the first thread in one group updates the probability at the state *PSR9_KMER_SKIP* for all blocks in this group, namely, fm[row][NUM_STATES*b + KMER_SKIP] (*b *=* *0 to *n_block*), and NUM_STATES * b is the offset for the current block. Finally, the log probability for the end state is obtained using the *add_log* function, which adds the log-scaled values.

Algorithm 2: Fill_kernel

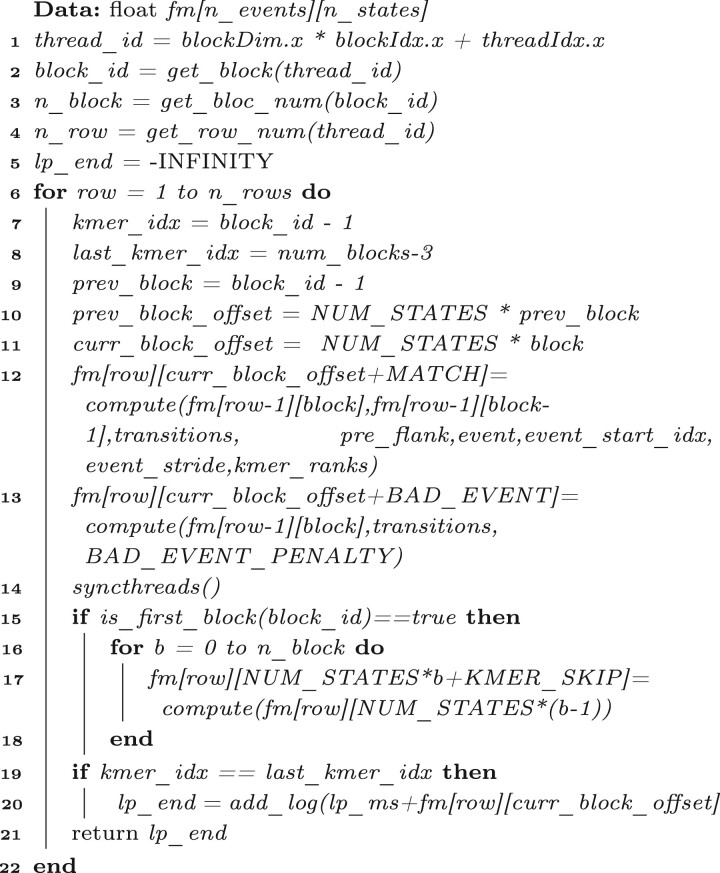

### 3.4 Pipelining

In the original *Nanopolish* call-methylation module, each batch of data is processed independently and every task is performed serially on each batch. So, when we add GPU parallelization to the methylation score calculation step, only during that task, the GPU utilization becomes very high and sometimes reaching to the point of GPU overutilization (e.g. out of memory). As a result, CPU underutilization happens during the GPU-parallelized task execution. On the other hand, during dataset loading and other similar tasks that require CPU-only execution, GPU resources are underutilized. This is caused by the interleaving between the CPU and the GPU.

**Fig. 3.**
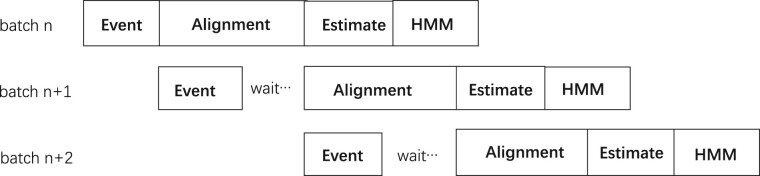
Methylation module’s four steps work in a pipeline fashion on three batches

To solve this issue of overutilization or underutilization of the CPU and GPU resources, we propose *Pipeline Optimization*, which uses heterogeneous processing. As illustrated in Figure 3, in this optimization, three batches are processed in a pipelined fashion—batch *n* is in the third step (final calibration), batch *n* + 1 is in the second step (alignment) and batch *n* + 2 is in the first step (event detection). To make sure that there are available resources, the next batch has to wait until the previous one is done (and it then performs the next step). For example, batch *n* + 1 has to wait till batch *n* finishes the alignment step, and then it performs the alignment step on GPU. This allows different batches to be processed *concurrently* and *heterogeneously*, which in turn improves the total performance and makes full use of the available hardware resources.

Synchronization is also important in pipeline optimization. In our pipeline implementation, one thread is launched to process a task of a batch. To ensure that enough resources are available, the thread has to wait for the completion of the previous step and starts the same step on the current batch. Figure 3 illustrates that, after the event detection, the execution waits for the completion of alignment on the previous batch and starts this step on the current batch.

The batch count is also a critical parameter that affects the pipeline behavior and overall performance. Since there are four steps to be done for each batch, the maximum concurrency is three parallel executed batches. If the batch count is too small (e.g. 2), the maximum concurrency cannot be achieved (causing underutilization). On the other hand, if the batch count is too large, the data loading time for the batches will delay the execution time significantly. For example, if we look closely at a timeline with batch counts of 3 and 12, we can observe that batches of data are loaded from the disk and then these batches are processed in the pipeline. Compared with the pipelined execution with a batch count of 3, the high data loading latency for execution with a batch count of 12 increases the end-to-end execution time significantly since more batches need to be loaded serially. For example, the data time resulting from loading batch 12 to batch 23 would delay the batch 12 processing time, and as a result, batch 12 will be processed at a much later time (after approximately 15 s for our experiments) than its loading time compared with that of batch number 3. In contrast, batch 12 starts to be processed only in a short amount of time after being loaded (approximately 5 s for our experiments). Setting the batch count to 3 leads to the best performance, as it achieves the maximum concurrency and the data loading does not delay the pipeline execution.

### 3.5 Adding *galaxy-methyl* into galaxy

We add *Galaxy-methyl* into Galaxy as a ‘tool’ for enabling researchers to take advantage of easy and fast methylation detection. In order to do that, we need to create a definition file for *Galaxy-methyl*, point to *Galaxy-methyl’*s path in that definition file and add a new section in Galaxy’s tool configuration file to let the Galaxy framework (Community, 2022) be aware of the new tool. The details of this process are as follows.

We created a definition file of *Galaxy-methyl* within the *Galaxy-methyl* directory to specify the way to execute the tool and pointed to *Galaxy-methyl*’s executable path within that definition file. The definition file is as Algorithm 3 shows. Line 3 specifies the command to execute the tool, the options to be passed and the files to be generated as the results. The input tag in lines 4–8 includes all parameters that are used in the command content, and their type and format are defined. When users use the tool from Galaxy platform, they fill these input parameters. Line 9 defines the output data generated by the tool as results. Finally, adding a new section in *tool_conf.xml* file within *config* directory lets the Galaxy framework to be aware of the new tool.

Algorithm 3: Definition file

1 <tool id=”methyl” name=”Galaxy-methyl” version=”v1.0”>

**2** <description>GPU accelerated and pipelined call-methylation module</description>

3 <command detect_errors=”exit_code”><![CDATA [$_ _tool_directory_ _/Galaxy-methyl call-methylation -t $threads -B $batch -r $read -b $bam -g $ref > ’$out_file1’]]></command>

4 <inputs><param type=”text” name=”threads” label=”number of threads”/>

5 <param type=”text” name=”batch” label=”size of batch”/>

6 <param type=”data” name=”read” format=”fastq.gz” label=”reads data”/>

7 <param type=”data” name=”bam” format=”bam” label=”alignments”/>

8 <param type=”data” name=”ref” format=”fasta” label=”reference genome”/></inputs>

9 <outputs> <data name=”out_file” format=”tsv”/> </outputs> </tool>

### 3.6 Experimental evaluation

We have added *Galaxy-methyl*, as a new tool, into the Galaxy platform (Community, 2022). Galaxy (https://galaxyproject.org/galaxy-project/statistics/) is a popular web-based platform for reproducing bioinformatics experiments with ready-to-use computational tools. In this work, we use the GPU-supported version of the Galaxy platform presented in Gudukbay *et al.* (2021). We also containerized *Galaxy-methyl* by creating an NVIDIA-Docker container, which is available at the Docker repository *gulsumgudukbay/galaxy-methyl* (https://hub.docker.com/r/gulsumgudukbay/galaxy-methyl). Our Docker container performs the same as the bare-metal execution excluding the container startup overheads. We have also compared the performance of *Galaxy-methyl* against the previous work. The call-methylation module of *Nanopolish v0.14.0* (our baseline) is a CPU-only implementation with multithreading, and the previous work *f5c v0.8* accelerates the ABEA algorithm with GPUs based on *Nanopolish*.

### 3.7 Platforms and setup

We have used two servers to conduct our experiments. Relatively speaking, Server_small_ has a higher number of CPUs and smaller GPU memory capacity, whereas Server_large_ has a larger GPU memory capacity and lower CPU count. More specifically, Server_small_ has a Xeon E5-2620 processor with 12 CPUs and 1 Tesla K20m GPU with 5 GB memory, and Server_large_ has a Xeon E5-2686 processor with 2 cores and 1 Tesla K80 GPU with 12 GB memory. The GCC 5.4.0 is used in compiling our application programs and the GPU driver version is 418.87 for both the servers used. The CUDA version is 8.0.

To test the effectiveness of our proposed approach, we use datasets from two different projects from European Nucleotide Archive (https://www.ebi.ac.uk/ena/browser/home) under accession numbers PRJEB23027 and PRJEB13021. The first project (PRJEB23027) contains Nanopore WGS Consortium (Jain *et al.*, 2018) sequencing data, where nanopore reads of the human genome (cell line NA12878) from 53 individual flow cells are available. Among 53, we chose reads from three individual flowcells under accession numbers ERR2184700, ERR2184710 and ERR2184719 and label them as *D*_1_, *D*_2_ and *D*_3_, respectively. From the second project (PRJEB13021), we choose three datasets under accession numbers ERR1676719, ERR1676724 and ERR167672 and label them as *D*_4_, *D*_5_ and *D*_6_, respectively. *D*_4_ contains nanopore reads from *Escherichia coli* strain K12 ER2925 whereas *D*_5_ and *D*_6_ contain reads from human cell line NA12878. We chose these six datasets because they have also been used in recent studies (Gamaarachchi *et al.*, 2020; Simpson *et al.*, 2017). Their salient features are listed in Table 2.

**Table 2.** Datasets

Dataset	*D*_1_	*D*_2_	*D*_3_	*D*_4_	*D*_5_	*D*_6_
Number of reads	668 016	117 140	38 335	88 328	91 579	28 169
Total bases	3203M	696M	190M	471M	274M	136M

We have designed different experiments to quantify the effectiveness of our approach—*Galaxy-methyl*. We have executed the call-methylation module with different datasets on Server_small_ and Server_large_ to show that *Galaxy-methyl* can be executed on different hardware configurations and how the performance varies across different CPU, GPU and memory configurations. The CPU version executes with the maximum supported threads on both the servers used. The maximum number of threads for Server_small_ and Server_large_ is 24 and 4, respectively. The execution time that we have measured includes the processing time for the methylation score calculation step and the total processing time of the call-methylation module. Note that the total processing time excludes the disk I/O processing time.

## 4 Results

**Fig. 4.**
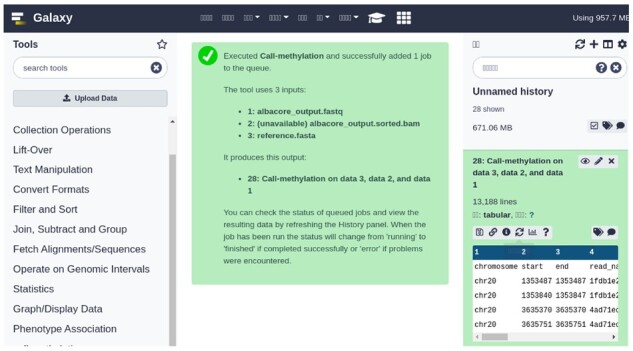
Galaxy interface to our tool

We have executed *Galaxy-methyl* in the Galaxy platform as an external tool. Figure 4 shows the result of *Galaxy-methyl* execution on Galaxy. The right part in the figure is the output.

We have also containerized *Galaxy-methyl* by creating an NVIDIA-Docker container, which allows lightweight portability and easy execution of the tool. The containerized version performs the same with the bare-metal execution excluding the Docker container startup overhead, which was approximately 20% of the actual runtime. However, this is not a problem, because the main reasons to adopt a containerized version are portability and easy execution (by reducing installation overheads). We have also checked the correctness of our *Galaxy-methyl* implementation by comparing the outputs of *Nanopolish* and *Galaxy-methyl*. Specifically, we have verified that the results of the log-likelihood ratio to each methylated and unmethylated CpG site group in *Nanopolish* are the same as that in our work for the example dataset.

We have compared the call-methylation module execution time of *Nanopolish*, *f5c* and *Galaxy-methyl* on the servers described above. We have first tested the performance of our GPU implementation of the methylation score calculation step (HMM) in *Galaxy-methyl*. Note that, in our measurements, the execution time of our GPU implementation includes the CUDA kernel execution time as well as the CPU–GPU data transferring time, but it does not include the array flattening time, which transforms some complex data structures to arrays supported in the kernel-side. We have used *Nvprof* to measure the latencies for CUDA kernel execution and data transfer between CPU and GPU. Then, we have measured and compared the total execution time of the call-methylation module.

**Fig. 5.**
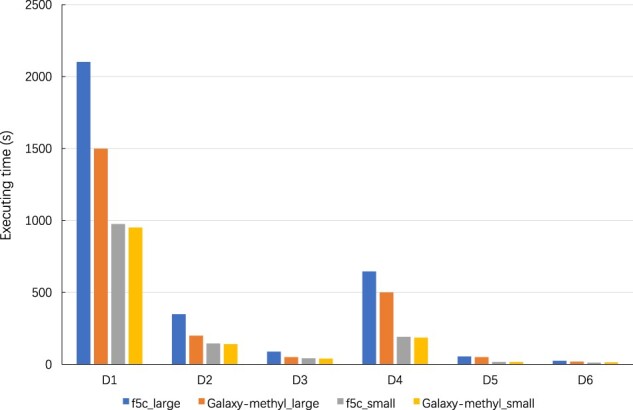
Methylation score calculation execution time comparison on Server_small_ and Server_large_

Figure 5 presents the methylation score calculation execution time achieved by our GPU implementation in *Galaxy-methyl* and that of the CPU-based implementation (OpenMP) in *f5c* on Server_large_ and Server_small_. One can observe that our GPU implementation of the methylation score calculation decreases the execution time by 35%, on average, compared with the CPU-based multi-threaded implementation of *f5c*. Our GPU implementation can achieve better speedup on datasets $D_{1}\;D_{2}$ and *D*_3_, where methylation score calculation is more compute-intensive. The figure also indicates that the methylation score calculation times for *f5c* and *Galaxy-methyl* are approximately the same on Server_small_, since the K20m GPU on Server_small_ has only 5 GB memory and can only process smaller batches. This leads to lower GPU utilization.

**Fig. 6.**
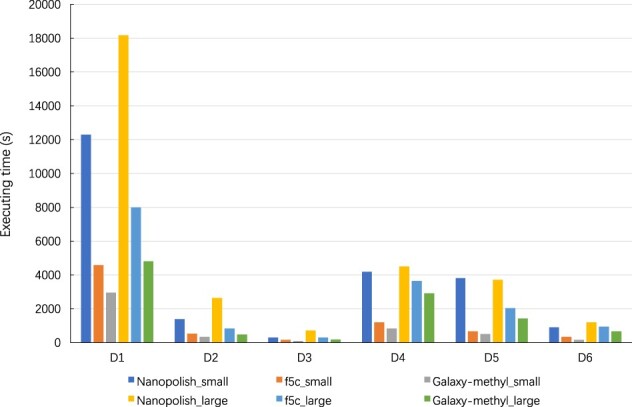
Call-methylation module execution time comparison on Server_small_ and Server_large_

Figure 6 compares the total call-methylation module latencies of *Galaxy-methyl*, *f5c* and *Nanopolish* on Server_small_ and Server_large_. *Galaxy-methyl* allows three batches to work in a pipelined fashion each time. It can be seen that, *Galaxy-methyl* decreases total processing time by more than 36%, on average, compared with *f5c*, and achieves 4.8× speedup compared with *Nanopolish* on Server_small_ on average. Furthermore, the results also reveal that *Galaxy-methyl* decreases the processing time by 34%, on average, with respect to *f5c*, and achieves about 3.2× speedup with *Nanopolish* on Server_large_ on average. These results also show that ABEA dominates the total processing time for *Galaxy-methyl* since it is the most time-consuming step in the pipeline.

Table 3 gives the memory consumption, sm_efficiency (percentage of time at least one warp is active on any multiprocessor, averaged over all multiprocessors) and achieved_occupancy (ratio of the average active warps per active cycle to the maximum number of warps supported on a multiprocessor) for our GPU implementation of methylation score calculation step on Server_small_ and Server_large_. We measure the average value for the one-time processing of three batches. The high sm_efficiency and achieved_occupancy values indicate that our proposed GPU implementation achieves high parallelism and high efficiency. Since K80 on Server_large_ has a larger memory size than K20m on Server_small_, the batch size is larger on Server_large_, which leads to higher memory consumption, sm_efficiency and achieved_occupancy. On average, sm_efficiency is 78% on Server_small_ and 90% on Server_large_. Additionally, achieve_occupancy reaches 0.6 on Server_small_ and 0.8 on Server_large_. So, it achieves better speedup on Server_large_.

**Table 3.** Memory consumption and GPU utilization on Server_small_ (*S*_s_) and Server_large_ (*S*_l_)

Dataset	Memory(MB)		Sm_efficiency (%)		Achieved_occupancy	
	*S*_s_	*S*_l_	*S*_s_	*S*_l_	*S*_s_	*S*_l_
*D*_1_	570	870	76	98	0.6	0.79
*D*_2_	457	774	79	99	0.6	0.81
*D*_3_	388	562	76	84	0.6	0.79
*D*_4_	558	830	97	99	0.8	0.9
*D*_5_	234	365	73	80	0.56	0.75
*D*_6_	255	390	68	75	0.53	0.72

**Fig. 7.**
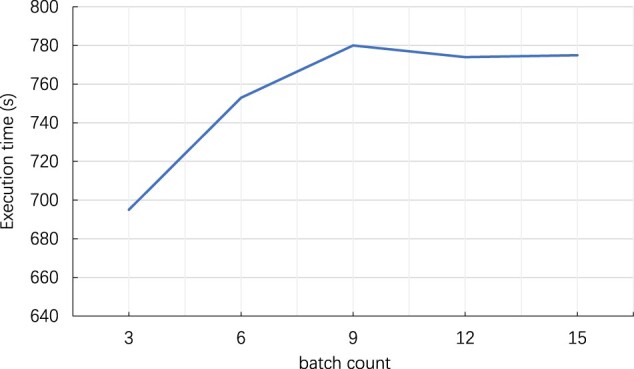
Execution time results with varying batch counts

We have also studied the impact of batch count on *Galaxy-methyl*’s performance. Figure 7 plots the execution time of *D*_6_ on server_large_ with various pipeline batch counts (which indicates how many batches are working in the pipeline each time). The batch count is increased from 3 to 15, and the results indicate that *Galaxy-methyl* can achieve the best performance when the batch count is 3, which is consistent with our pipeline optimization approach described in Section 3.4.

## 5 Conclusion

Our work parallelizes and optimizes methylation score calculation step, which is the major performance bottleneck in *Nanopolish* on emerging GPU platforms. Our work also pipelines all four steps of the module to make full use of hardware resources in the underlying compute platform. The collected experimental results reveal that our GPU implementation of the methylation score calculation step can achieve ∼2× speedup compared with the CPU-only implementation. Further, our implementation can also achieve 2× ∼3.2× speedup compared with *Nanopolish*, and reduce 34%–52% of the total execution time of *f5c*.

## Data Availability

The input data that we used to experiment with Galaxy-Methyl are available in European Nucleotide Archive (ENA) at https://www.ebi.ac.uk/ena/browser/home, and can be accessed with accession PRJEB23027 and PRJEB13021. The raw performace data generated by experiments are available at https://github.com/fengyilin118/Galaxy-methyl/tree/main/data.
